# Effects of Passive Movement on Motor Function and Disability in Patients with Stroke: A Systematic Review and Meta-Analysis

**DOI:** 10.3390/jfmk10020117

**Published:** 2025-03-31

**Authors:** Auwal Abdullahi, Thomson W. L. Wong, Shamay S. M. Ng

**Affiliations:** Department of Rehabilitation Sciences, The Hong Kong Polytechnic University, Hong Kong Special Administrative Region, China; aabdullahi.pth@buk.edu.ng (A.A.); thomson.wong@polyu.edu.hk (T.W.L.W.)

**Keywords:** stroke, passive movement, motor impairment, disability, quality of life

## Abstract

**Background**: Severe impairment in motor function following a stroke can pose a significant challenge during rehabilitation since the patients are unable to carry out active forms of rehabilitation, such as task-specific training. Thus, in such a case, passive movement can be utilized. The aim of this systematic review and meta-analysis is to determine from the literature the evidence on the effects of passive movement compared with a control on recovery outcomes post stroke. **Method**: Four databases, PubMED, Embase, Web of Science (WoS), and CENTRAL, were searched. Data on the study participants’ characteristics, such as the mean age, the mean time since stroke, the protocol of the experimental and control interventions, the mean scores on the outcomes assessed post intervention and at follow-up, and the number of participants in both the experimental and control groups were extracted. **Result:** Four studies (n = 166), with two having a moderate quality and two having a high methodological quality, were included in the study. The test for overall effects showed that passive movement is superior to the control at improving the recovery of function (SMD = 0.82, 95% CI = 0.40 to −1.24, *p* = 0.0002) post intervention. However, the results of the individual domains showed that the experimental group is only superior to the control at improving motor function (SMD = 0.70, 95% CI = 0.21 to 1.18, *p* = 0.005) and disability (SMD = 0.81, 95% CI = 0.32 to 1.31, *p* = 0.001). **Conclusions:** Evidence for the effects of passive movement on recovery outcomes in patients with stroke seems to be low. Therefore, the clinical decision on its application requires reflection, and further randomized controlled trials need to be carried out to determine the evidence.

## 1. Introduction

Stroke is a condition that can affect a patients’ ability to move [1]. Ability to move, for instance, the upper and lower limbs is essential for carrying out the activities of daily living (ADL) [2]. However, it is important to note that following a stroke, the ability to move any part of the body, such as the limbs, may depend on the degree of the residual paralysis and motor impairment [1]. Thus, depending on this degree of severity, a particular rehabilitation can be employed. For instance, in patients whose degree or level of impairment is mild to moderate impairment, active techniques of rehabilitation, such as task-specific training or constraint-induced movement therapy (CIMT), are used [2,3,4,5,6,7]. For those with severe impairment, the passive forms of these techniques, such as passive movement or mobilization, mental practice and task observation (motor imagery), non-invasive brain stimulation, and neurodevelopmental therapy (NDT), are used [8,9,10,11,12,13,14]. These techniques are all passive methods of rehabilitation that do not require the active performance of tasks with the affected limb.

The problem with some of these passive techniques is that their use is usually limited. For example, mental practice and task observation (motor imagery) cannot be performed if the patients have severe impairment in cognitive function [15]. Similarly, when patients have cardiac pace makers or implants or certain serious medical conditions such as epilepsy, techniques such as neuromuscular electrical stimulation and non-invasive brain stimulation cannot be employed [16]. In addition, acquiring the devices used for the aforementioned techniques can be very costly. However, one of the techniques, passive movement, is easy to administer and can be carried out even when the patients have severe impairment in cognitive function or are unconscious. Furthermore, it is not costly to administer passive movement as it can be administered manually without any equipment.

Passive movement is the manipulation of a body part or limb without the voluntary effort of the patient or the individual [17]. It is said to induce cortical activity, although to lesser extent compared to active movement [18]. This study aims to determine the current available evidence on the effects of passive movement on recovery outcomes in patients with stroke. Consequently, we hypothesized that passive movement will not be effective at improving recovery outcomes in patients with stroke.

## 2. Method

This systematic review and meta-analysis was carried out according to the Preferred Reporting Items for Systematic Reviews and Meta-Analyses (PRISMA) guidelines [19] and was registered in the International Prospective Register of Systematic Reviews (PROSPERO) with the registration number CRD42023464706.

### 2.1. Inclusion and Exclusion Criteria

Participants were included based on PICOS (population, intervention, comparator/control, outcome, and study design) criteria. The population included patients with stroke who were 18 years old and above. The intervention was passive movement. The comparator/control was any intervention or sham other than passive movement. The outcome was recovery outcomes, such as motor function and disability. The study design was a randomized controlled trial (RCT). In addition, only studies published in English were included. See Table 1 for the selection criteria according to the PICOS criteria.

### 2.2. Searching the Literature

In the study, the databases searched were PubMED, Embase, the Web of Science (WoS), and the Cochrane Central Register of Controlled Trials (CENTRAL). The search was carried out from the earliest dates of the databases to January, 2025. To make the search comprehensive, an additional manual search of the lists of references of previous relevant systematic reviews and meta-analyses was carried out.

The search terms used include stroke, range of motion, joint range of motion, and exercise, with their respective Medical Subject Headings terms. Details of the strategies used for each of the databases are presented in Appendix A. The search was done independently by one of the researchers, (AA); however, it was carefully scrutinized by the other researchers (TWLW and SSMN).

Finally, the search was entered into Endnote software (version 21), which was used to remove duplicate articles.

### 2.3. Selection of Eligible Studies and Data Extraction

The selection of eligible studies started from excluding studies based on the contents in their titles and abstracts. Following this, full texts of the remaining studies were read to ascertain whether they were eligible.

The process of the selection was independently carried out by two of the researchers (AA and TWLW). However, they met afterwards to achieve consensus on the selection, and if there was any dispute, they involved the third researcher (SSMN).

Similarly, one of the researchers (AA) extracted data on the characteristics of the participants included in the studies, such as their mean age, time since stroke, type of stroke, side affected, the interventions used in the experimental and the control groups, the outcomes assessed such as motor function, level of motor impairment, ADL, and quality of life, their mean scores post intervention and at follow-up, and the study sample sizes. The extracted data were then scrutinized by the other researchers (TWLW and SSMN) for quality assurance.

PRISMA flowchart was used to summarize the study selection process.

### 2.4. Risk of Bias and Methodological Quality Assessments of the Included Studies

The risk of bias of the included studies was assessed using the Cochrane Risk of Bias Assessment tool. Similarly, the methodological quality of the included studies was assessed using the PEDro scale.

The Cochrane Risk of Bias Assessment tool assesses potential biases in participants’ selection, allocation concealment, blinding of study participants and personnel, blinding of outcome assessors, handling of participants attrition, reporting of results, and other possible biases that might occur during the conduct of the study [20].

The PEDro scale assesses the internal and external validity of the RCT. It comprises of eleven items, in which the first one assesses the external validity of the studies, whereas the remaining ten items assess their internal validity [21]. The items that assess the internal validity are further rated on a two-point scale that ranges from 0 (no to the question in the item) to 1 (yes to the question in the item). Thus, the total score for the internal validity items range from zero to ten; and it is interpreted as being of low, moderate, and high methodological quality when it is 0 to 3, 4 to 5, and 6 to 10 respectively [22,23,24].

Both assessments were performed by two of the researchers (AA and TWLW). Following this, the two researchers sat together to agree on their selections; where possible, they also involved the third researcher (SSMN) to resolve any disputes that might arise.

### 2.5. Data Synthesis

Narrative and quantitative syntheses were used for the analysis of the extracted data. For the narrative synthesis, a summary of the characteristics, risks of bias, and methodological quality of the included studies was carried out. In the quantitative synthesis, a random effect model meta-analysis was performed by pooling the mean scores and standard deviation of the outcomes of interest and the number of participants in the studies post intervention and at follow-up to determine the overall mean difference between the experimental and control group. However, it is important to note that only outcomes that were reported in at least two studies were analyzed.

In addition, the percentage of variation across the studies due to heterogeneity was determined using (*I*^2^) statistics; it was judged to be significant when its value was between 50 and 90% at *p* < 0.05.

The quantitative synthesis was carried out using RevMan software (version 5.4).

The results were presented in tables and figures.

### 2.6. The Interpretation of the Evidence from the Findings

The evidence from the findings of this study was interpreted using GRADE (the grading of recommendations, assessment, development, and evaluation instruments) [25]. It is an instrument that consists of the risks of bias, imprecision, inconsistency, indirectness and publication bias as domains.

## 3. Results

### 3.1. Narrative Synthesis

#### 3.1.1. Selection of Eligible Studies

**Figure 1:**
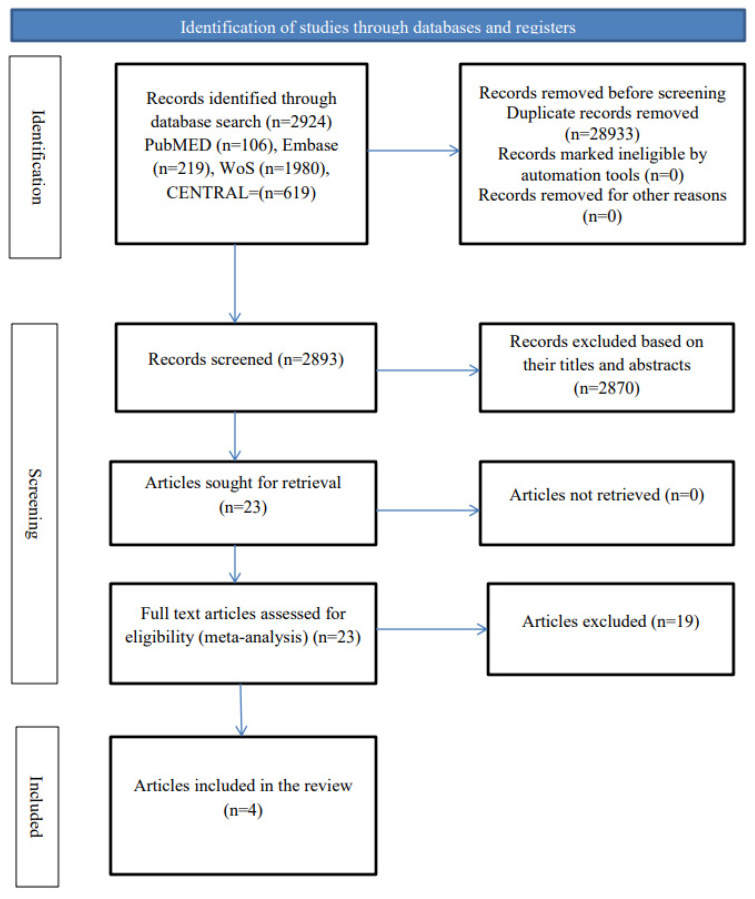
Study Flow chart.

The search of the databases produced a total of 2924 articles, in which only 4 studies were finally deemed eligible and were included in the study [26,27,28,29]. However, some potentially eligible studies were excluded because they were quasi-experimental studies [30,31] or involved the use of both active and passive movement [32,33]. The process of the search and the subsequent selection of the studies are represented in Figure 1.

#### 3.1.2. The Characteristics of the Included Studies

The included studies had a total sample size of 166 patients with stroke (range, 32 to 52), mean age range, 59.2 ± 14.1 to 90 years. However, only one study provided information on the mean time since stroke, which was 12.0 + 2.0 days to 14.0 + 2.0 days [26]. Out of the above total number of participants, 72 were female. The pathological types of stroke that the patients had were only reported in three studies, comprising 96 ischaemic and 25 hemorrhagic [26,27,28]. Similarly, only six studies reported the sides affected, which were 77 right- and 89 left-side hemiplegia.

Only one study did not provide information on the stroke phase of the included participants at the time of enrolment [26]. Two studies enrolled participants during the acute stage [27,28]; one study enrolled participants during the chronic stage [33].

Two studies included participants with a severe impairment or disability; a score of <20 upon upper-limb Fugl Meyer motor assessment [26]; and recovery stages 1 and 2 as per the Bruunstrom recovery stages [27]. All the remaining studies included participants with either a mild or moderate disability or a mild, moderate, or severe disability. However, the participants were excluded if they had a significant cognitive impairment [26,27,28,29], orthopedic problem, or joint deformity before the stroke [26,27,28,29]; any existing medical condition that may contraindicate medical treatment [27,28]; or joint pain [29].

The outcomes assessed in the studies include the level of motor impairment [22]; motor function [26,27]; muscle strength [26,27,28,29]; joint range of motion [27,29]; disability [26,27]; joint pain [26]; shoulder joint stability [26]; muscle tone [26]; gait velocity [29]; stride length [29]; cadence [29]; and oedema [27]. Further characteristics of the study participants are presented in Table 2.

#### 3.1.3. Methodological Quality and Risks of Bias

**Figure 2:**
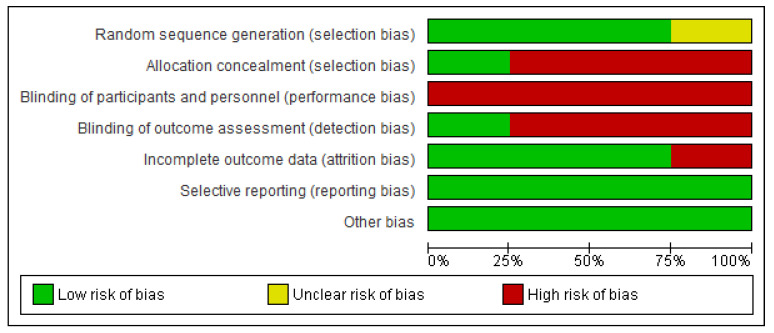
The risk of bias graph of the included studies.

Two of the included studies had a moderate methodological quality [26,28], whereas the remaining two had a high methodological quality [27,29]. See Table 3 for more details. However, some of the studies had high risks of bias in allocation concealment [26,27,29]; outcome assessment [26,28,29]; personnel and subjects blinding [26,27,28,29]; and attrition [26]. In addition, one of the studies had unclear risks of bias in random sequence generation [26]. See Figure 2 for the risk of bias graph.

### 3.2. Quantitative Synthesis

Only three studies were included in the quantitative synthesis.

#### 3.2.1. Recovery of Function

At post intervention, the test for overall effects indicated that participants in the experimental group significantly regained function compared to the control (SMD = 0.82, 95% CI = 0.40 to 1.24, *p* = 0.0002).

**Figure 3:**
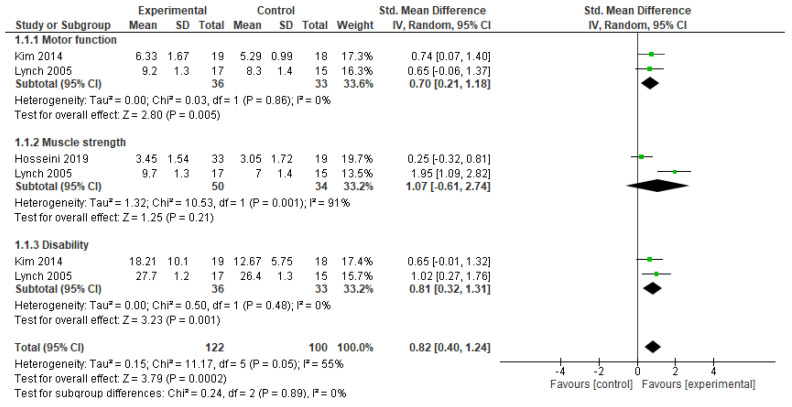
Effects of passive movement on outcomes of interest.

However, for the individual domains of the recovery of function, the experimental group was only superior to the control at improving motor function (SMD = 0.70, 95% CI = 0.21 to 1.18, *p* = 0.005) and disability (SMD = 0.81, 95% CI = 0.32 to 1.31, *p* = 0.001). In addition, there was no significant heterogeneity between the included studies for the motor function (I2 = 0%, *p* = 0.86) and the disability (I2 = 0%, *p* = 0.48) outcomes. See Figure 3 for the details of the results.

For muscle strength, although there was no significant difference between the groups, there was a trend towards more significant improvement in the experimental group (SMD = 1.07, 95% CI = ÷0.61 to 2.74, *p* = 0.21), with significant heterogeneity between the included studies (I2 = 91%, *p* = 0.001). See Figure 3 for the details of the results.

#### 3.2.2. The Interpretation of the Evidence

The evidence for the effects of passive movement on recovery outcomes in patients with stroke seems to be low. See Table 4 for the quality assessment of the evidence. Therefore, more RCTs need to be carried out to further determine the evidence.

## 4. Discussion

The aim of this study was to determine the current available evidence on the effects of passive movement on the recovery outcomes in patients with stroke compared to the control. The result showed that overall, passive movement is superior at improving recovery of function compared to the control. Thus, the findings contradict our hypothesis that passive movement will not be effective at improving recovery outcomes in patients with stroke. However, based on the specific domains, it is only superior to the control at improving motor function and disability. This could be because passive movement induces cortical activity, though not as strongly as active movement [18,34,35]. Increased cortical activity results in improvement in movement ability, which is positively associated with reduced disability [1,36].

In addition, passive movement can produce several effects in patients with stroke. It can enhance metabolic oxygenation, which will in turn help prevent the metabolic deterioration of the muscles [37]. Metabolic deterioration is a precursor to reduced motor function and muscle strength. Similarly, passive movement can also improve spasticity and functional ambulation [38]. Improvement in spasticity and functional ambulation can enhance patients’ ability to carry out ADL and increase their quality of life. However, one of the concerns on the use of passive movement in stroke rehabilitation is the mode of the delivery of the intervention. In some of the studies, the movement was generated from the efforts of the clinician [27,28,29]; whereas, in one of the studies, it was generated by a machine [26]. Consequently, it has been argued that in the course of carrying out passive movement, the stimuli generated by machine or device is uniform in contrast with the one generated by the clinicians [31].

Moreover, unlike the machine, the clinicians that carry out the passive movement may suffer fatigue that can affect their physical and mental states, which may in turn affect the intervention negatively [39]. In contrast, with the machine, the same intensity, frequency, and range can be used throughout the intervention period [31]. Thus, future studies need to compare the effects of using manual and mechanically delivered passive movement. Furthermore, since passive movement is not as potent as active movement in inducing cortical activity post stroke, the high repetition of passive movement may be necessary for the optimal induction of cortical activity. This is because, even with the use of active movement, high repetition is required to induce cortical activation and the subsequent recovery of function [2,40,41].

Similarly, the included studies are not clear about the intensity of the interventions used in the experimental and the control groups. For instance, in one of the studies, information on the duration of the interventions in both groups was not provided [26]. Secondly, the daily intensity of the protocol in the control group was not provided. In addition, there was variation in the protocols of the experimental and control groups, the outcome measures used, and the part of the body treated. Thus, future studies should standardize the protocols in terms of the intensity and other parameters of passive movement they use.

Although this study may have some peculiar limitations, one of its strengths is that the literature was searched comprehensively using major databases. A comprehensive literature search is a key step in carrying out valid and reliable research [42]. Similarly, a major weakness of this study is the lack of sensitivity analysis due to the limited number of the included studies. In addition, the included studies had small sizes and significant heterogeneity between them. Studies with small sizes often show different and large effects compared to those with large samples, and as such, it is difficult for them to detect true effects [43]. Moreover, statistical heterogeneity indicates inconsistency between studies for example in the use of outcome measures [44]. Thus, it is important that future studies use standardized outcome measures and adequate sample sizes to determine the effects of passive movement on recovery outcomes in patients with stroke.

## 5. Conclusions

The evidence for the effects of passive movement on recovery outcomes in patients with stroke seems to be limited. Thus, the clinical decision on its application requires reflection. In addition, more randomized controlled trials need to be carried out to determine the evidence. The studies should use adequate sample sizes and standardized outcome measures to determine the effects of passive movement on recovery outcomes in patients with stroke.

## Figures and Tables

**Table 1:** Selection criteria used according to PICOS model.

PICOS Category	Inclusion Criteria	Exclusion Criteria
P (population)	Studies on males or females with stroke who are 18 years or older, within any stage of stroke	Studies not on patients with stroke
I (intervention)	Passive movement of joints	Unclearly described passive movement
C (comparators)	Any intervention or sham other than passive movement	Unclearly described intervention
O (outcomes)	Recovery outcomes such as motor function and disability	Outcomes that are not reported to be valid and reliable
S (study design)	Randomized controlled trial (RCT)	Study designs other than RCTs

**Table 2:** Characteristics of the included studies.

References	N	Stroke Duration	Mean Age (Years)	Intervention	Outcomes	Findings	Adverse Events
Lynch et al. [26]	N = 32; experimental (n = 17, females = 12); control (n = 15, females = 7)	Experimental = 14.0 + 2.0 days; control = 12.0 + 2.0 days	Experimental = 61.0 + 3.0; control = 71.0 + 3.0	Participants in both groups received standard interdisciplinary therapy for 3.5 hrs per day. Experimental = received 25 min daily continuous passive motion comprising shoulder elevation, external rotation, and abduction, 5 times a week. Control 2 = performed group self-range of motion focused on goal-directed movements of the shoulder.	Level of motor impairment (FMA); motor function of shoulder and elbow (MSS); muscle power of wrist and hand (MPS); disability (FIM); joint pain (FMA pain index); shoulder joint stability; muscle tone (MAS).	No significant difference between groups in the outcomes of interest post intervention (at discharge).	Not reported
Kim et al. [27]	N = 37 experimental (n = 19, females = 5); control (n = 18, females = 10)	Not reported	Experimental = 59.2 ± 14.1; control = 63.0 ± 16.2	Experimental = performed 15 min bilateral passive ROM exercise of the upper limb joints twice a day, 5 days per week for 4 weeks. Each movement was repeated 10 times during the session. Control = performed same exercise as in the experimental group, but only after 2 weeks.	Upper extremity oedema (tape measure); ROM (goniometry); motor function (MFT); disability	All outcomes improved more significantly in the experimental group compared to the control post intervention.	Not reported
Hosseini et al. [28]	N = 52; experimental (n = 33, females = 16); control (n = 19, females = 10)	Not reported	Experimental = 30–90; control = 30–90	Experimental = performed 15–40 min passive ROM exercise of the upper limb joints 6 to 8 times. Control = received only routine care during the period.	Muscle strength (MRC scale).	Significant improvement in muscle strength in the experimental group.	Not reported
Cho and Park [29]	N = 45; experimental 1 (n = 15, females = 5); experimental 2 (n = 15, females = 5); control (n = 15, females = 2)		Experimental 1 = 64.53 + 8.05; experimental 2 = 64.66 + 5.32; control = 63.40 + 7.09	Experimental 1 = 3rd stage of the joint mobilization (gliding) was performed to induce dorsiflexion of the ankle joint repeatedly for 15 min. Control = performed active stretching of the ankle joint, repeated for 15 min. Experimental 2 = received a combined joint mobilization in experimental group 1 and the active stretching in the control group for 15 min. Intervention was done for 6 weeks.	ROM (goniometer); cadence, gait velocity and stride length (Gait–Walk).	All outcomes improved in the experimental groups. However, the improvement was more significant in the experimental group 2 (a combination of group experimental 1 and control group intervention).	Not reported

Key: FMA = Fugl Meyer motor assessment, MSS = motor status scale, MPS = motor power scale, FIM = Functional Independence Measure, MAS = modified Ashworth scale, ROM = range of motion, MFT = manual function test, MRC = Medical Research Council scale.

**Table 3:** Methodological quality of the included studies.

Study	Eligibility Criteria Specified	Random Allocation	Concealed Allocation	Comparable Subjects	Blind Subjects	Blind Therapists	Blind Assessors	Adequate Follow-Up	Intention to Treat Analysis	Between Group Comparison	Point Estimation and Variability	Total Score
Lynch et al. [26]	Yes	1	0	1	0	0	0	1	0	1	1	5/10
Kim et al. [27]	Yes	1	0	1	0	0	1	1	1	1	1	7/10
Hosseini et al. [28]	Yes	1	1	0	0	0	0	0	1	1	1	5/10
Cho and Park [29	Yes	1	0	1	0	0	1	1	1	1	1	7/10

**Table 4:** Evidence quality assessment.

						Number of Participants			
Outcome	Number of Studies	Risks of Bias	Inconsistency	Indirectness	Imprecision	Experimental	Control	Effect Size (95% CI)	Overall Certainty of the Evidence
Motor function	2	Serious	Very serious ^a^	Not serious	Serious ^b^	36	33	0.70 (0.21 to 1.18)	⨁⨁◯◯ Moderate
Muscle strength	2	Serious	Very serious ^a^	Not serious	Serious ^b^	50	34	1.07 (−0.61 to 2.74)	⨁⨁◯◯ Moderate
Disability	2	Serious	Very serious ^a^	Not serious	Serious ^b^	36	33	0.81 (0.32 to 1.31)	⨁⨁◯◯ Moderate

^a^ Significant heterogeneity; ^b^ sample size < 400.

## Data Availability

All the data used in this study are included within the manuscript.

## Appendix A

PubMED	(((((Stroke) OR (Ischaemic stroke)) OR (Hemorrhagic stroke)) OR (Brain infarction)) AND (Exercise)) AND ((((Range of motion) OR (passive range of motion)) OR (joint range of motion)) OR (joint flexibility)) Filters: Humans: English; RCTs; Adults
Author Contributions: WoS	(((((Stroke) OR (Ischaemic stroke)) OR (Hemorrhagic stroke)) OR (Brain infarction)) AND (Exercise)) AND ((((Range of motion) OR (passive range of motion)) OR (joint range of motion)) OR (joint flexibility)) Filters: Stroke; Rehabilitation; English; Trials; Web of Science citation index (expanded and emerging); WoS conference citation index
Embase	(((((Stroke) OR (Ischaemic stroke)) OR (Hemorrhagic stroke)) OR (Brain infarction)) AND (Exercise)) AND ((((Range of motion) OR (passive range of motion)) OR (joint range of motion)) OR (joint flexibility)) Filters: English; RCTs; cerebrovascular accident
CENTRAL	(((((Stroke) AND (Exercise)) AND ((((Range of motion) OR (passive range of motion)) OR (joint range of motion)) Filters: CINAHL; English; Trials
